# Give Up Flights? Psychological Predictors of Intentions and Policy Support to Reduce Air Travel

**DOI:** 10.3389/fpsyg.2022.926639

**Published:** 2022-08-04

**Authors:** Jessica M. Berneiser, Annalena C. Becker, Laura S. Loy

**Affiliations:** ^1^User Behavior and Field Trials, Fraunhofer Institute for Solar Energy Systems ISE, Freiburg, Germany; ^2^Institute of Psychology, Otto-von-Guericke-University Magdeburg, Magdeburg, Germany; ^3^Department of Psychology, University of Koblenz-Landau, Landau, Germany

**Keywords:** pro-environmental behavior, policy support, sustainable mobility, intergenerational justice, efficacy beliefs, perceived behavioral control

## Abstract

Concerted, timely action for mitigating climate change is of uttermost importance to keep global warming as close to 1.5°C as possible. Air traffic already plays a strong role in driving climate change and is projected to grow—with only limited technical potential for decarbonizing this means of transport. Therefore, it is desirable to minimize the expansion of air traffic or even facilitate a reduction in affluent countries. Effective policies and behavioral change, especially among frequent flyers, can help to lower greenhouse gas emissions. For both, a positive evaluation and public support is indispensable. This study contributes to understanding air travel behavior and the perception of regulative policies. We examined the role of attitudes, perceived behavioral control, efficacy, global identity, and justice concerns for intentions to avoid flights and aviation-related environmental policy support. We conducted an online survey study with a quota sample of *N* = 2,530 participants in Germany. The strongest positive predictors of intentions to refrain from flying and policy support were perceived behavioral control to travel without flying, efficacy beliefs that avoiding air travel contributes to climate change mitigation, and intergenerational justice concerns; pro-travel attitude was a negative predictor. Moreover, we tested whether the provision of additional information on climate impact, global and intranational inequalities as well as subsidies (implying intranational inequality) affected the intention to avoid air travel and policy support. We found no effects of the different types of information. Nor did we find an interaction between the type of information provided and global or national identity. Our results highlight the need for a shift within the mobility sector that facilitates attractive and accessible transport alternatives in order to strengthen people’s behavioral control to choose other means than planes and their efficacy perceptions. Moreover, raising awareness of the impacts of climate change on future generations and developing strategies to promote people’s concern for intergenerational justice might motivate people to reduce air travel and thereby contribute to a livable future for new generations.

## Introduction

The latest IPCC report states that human-induced climate change is progressing fast and with drastic, irreversible consequences that are already observable and measurable (IPCC, 2022a). Greenhouse gas savings need to be promoted in all sectors to achieve the Paris climate protection targets (UNFCCC, 2015; IPCC, 2018). Aviation is responsible for approximately 2–3% of annual global CO_2_ emissions with the non-CO_2_ emissions not yet accounted for (Klöwer et al., 2021; EASA, 2022). Lee et al. (2021) estimated that CO_2_-warming-equivalent emissions stemming from aviation are three times higher than merely considering CO_2_ emissions. Prognoses state that aviation emissions could account for 25% of the global carbon budget by 2050 (Graver et al., 2019).

Decarbonizing aviation is technically challenging and requires significant investments (Chiaramonti, 2019). Therefore, alongside the need to address these technical challenges, a change in mobility behavior to cut fuel demand is of particular importance (Davison et al., 2014; Transport and Environment, 2018). This is especially the case, since reductions in CO_2_ emissions through efficiency and fuel switch are predicted to be negated by growing demand (Kahn Ribeiro et al., 2007). According to Gössling and Humpe (2020), existing policy plans and mitigation targets (e.g., under the Kyoto Protocol and Paris Agreement) ignore a large part of emissions. In the realm of passenger flights, efforts are deployed to make tourism more sustainable and reduce greenhouse gas emissions from travel (Gössling, 2009). Aviation accounts for a majority of the tourism sector’s CO_2_ emissions (Gössling, 2009). Expanding environmentally friendly mobility alternatives (e.g., trains) and promoting sustainable travel choices can thus aid in curbing emissions and decarbonizing the transport sector.

Hence, changes on different levels are needed: on the technological, political but also individual level (Transport and Environment, 2018, 2021). The latter two are especially relevant from an individual and societal perspective. First, public support is advantageous for the implementation of effective policy measures to reduce air traffic. Examining factors that increase and maintain policy support will allow recommendations on how to promote a climate-friendly mobility infrastructure and its usage.

Second, planes are taken by people—the aggregate individual mobility decisions represent the demand for air transportation. The advent of the Fridays for Future movement sparked debate about the environmental impact of air travel. It culminated in media reports discussing the need for and prevalence of “flight shame” (Gössling et al., 2020). Understanding the determinants of people’s intentions to refrain from flying could contribute to lowering carbon emissions through encouraging behavioral change accordingly.

Third, accessible sustainable alternatives to air travel are needed. For example, switching to (night) trains instead of planes constitutes a relevant option for reducing emissions (European Environment Agency, 2020). Research on travel mode choices found that people, for example, consider travel time and costs (Román et al., 2007) and familiarity (Dällenbach, 2020). A recent study showed that the perceived behavioral control over choices between modes of transport was associated with reduced airplane usage (Dütschke et al., 2022).

Our study contributes to understanding people’s intentions and policy support to reduce flying by examining further potentially relevant psychological factors next to perceived behavioral control over travel options: people’s travel and environmentally careless holiday attitudes, their efficacy beliefs in positive outcomes of flight avoidance, concerns for intergenerational justice as well as their global identification.

From a societal point of view, air travel is related to various inequalities. First, air travel is linked to intragenerational inequalities in two regards. On a global level, more people take planes in the Global North vs. the Global South [e.g., Latin America and the Caribbean accounted for 5.1% of passenger kilometers in 2019, Africa for 2.1%, and Europe for 26.8%; ICAO (2019)]. At the same time, many countries of the Global South are disproportionately affected by climate change (IPCC, 2022a). On a national level, air travel is highly subsidized but only a small amount of people tends to fly on a regular basis and thus profits from these subsidies—usually with high socioeconomic status (Gössling and Humpe, 2020). Second, the consequences of climate change will be experienced mostly by younger and future generations implying intergenerational inequalities (Page, 1999; Fleurbaey et al., 2014). These inequalities, and whether people perceive them as injustices, have received little attention in previous research explaining individual behavior and policy support. Thus, we examined whether the salience of different forms of air travel-related inequalities influences people’s intentions to avoid flights and support for aviation-related policy measures. Our study was conducted in Germany, a high-income country with comparatively good infrastructure for air, rail, and car travel.

## Theoretical Background

### Air Travel as Environmentally Relevant Behavior

Within the last years, flying has received more attention as an impactful environmentally relevant behavior due to its relevance for climate change mitigation (Gössling and Humpe, 2020). Reflections about the freedom of choice and the necessity of traveling by plane will not be targeted in this paper [see Gössling et al. (2019), for a respective discussion]. Rather, we aim to understand people’s motivation to reduce air travel. According to Stern (2000), environmentally relevant behaviors can be differentiated in high vs. low impact behaviors and private vs. public sphere behaviors. With regard to the high impact behavior of air traveling, people can decide to privately reduce their behavior in the mobility system and to support certain public policies and regulatory frameworks that shape or change this mobility system (Steg and Vlek, 2009; Dekoninck and Schmuck, 2022).

In this study, we investigate determinants of both individual intentions to refrain from air travel as well as support for policies that encourage the reduction of air travel. Previous studies have identified various factors that influence individual mobility choices and support for environmental policies [for overviews, see Drews and van den Bergh (2016) and Lanzini and Khan (2017)]. In the following section, we derive the specific contribution of our study to this research field.

#### Socio-Economic Correlates of Air Travel Behavior

High income (Böhler et al., 2006; Büchs and Schnepf, 2013) as well as higher levels of education (Böhler et al., 2006) are generally correlated with individual greenhouse gas emissions. Moreover, socio-economic characteristics are relevant for explaining mobility patterns in general, and air travel in specific. For example, both income and level of education predicted the frequency and distance of long-distance trips (Holz-Rau et al., 2014; Reichert and Holz-Rau, 2015). Dütschke et al. (2022) found that income and education predicted past flying behavior, with education being the stronger predictor. Only education predicted behavioral intentions to reduce long-distance travel. Regarding policy support, socio-economic (and further demographic) factors appear to have only a minor explanatory value (Ejelöv and Nilsson, 2020). Kallbekken and Sælen (2021) infer from the limited evidence that findings are inconsistent but policy support seems to be typically stronger among people with higher income. In the present study, we therefore also examine the role of income and education.

#### Psychological Determinants of Air Travel Behavior

An abundance of research has identified psychological predictors of environmentally relevant behaviors in general [for an overview, see e.g., Bamberg and Möser (2007), Klöckner (2013), and Gifford and Nilsson (2014)] and mobility behaviors in particular (Lanzini and Khan, 2017). According to the widely applied Theory of Planned Behavior (TPB, Ajzen, 1991), attitude, subjective norms, and perceived behavioral control toward a specific behavior are important determinants of many (pro-environmental) behavioral intentions and behaviors [for a review and guidance on the application of the TPB to pro-environmental behavior, see Yuriev et al. (2020)].

Past research on air travel found that particularly differences in attitudes and perceived behavioral control explained differences in behavior. While a pro-travel attitude (i.e., valuing freedom of travel choices and opportunities to travel frequently) was positively related to air travel choices, pro-environmental holiday attitude was negatively related (i.e., caring about the environment when planning vacation, see Barr and Prillwitz, 2012). In a recent study, Dütschke et al. (2022) found that people who perceived a higher behavioral control to travel without flying were less likely to have used a plane in the past and reported higher intentions to use more sustainable travel modes than air travel in the future. Next to behavioral control, the extent to which people believe that their behavior effectively leads to desirable outcomes is considered to impact sustainable behavioral choices (Hanss and Böhm, 2010). Accordingly, Doran et al. (2015) found that efficacy beliefs about one’s own contribution to environmental protection predicted the willingness to accept economic sacrifices when choosing traveling options. This is relevant to refraining from air travel as flying is often cheaper than alternative transport modes (Carrington, 2021). In accordance with the TPB, Davison et al. (2014) found that subjective social norms, but also personal norms [see e.g., Norm Activation Model, Schwartz (1977) and Schwartz and Howard (1981)], predicted the selection of alternatives to air travel and flight frequency. Contrarily, Dütschke et al. (2022) found no association between social and personal norms with intentions to refrain from flying. In our study, we focus on pro-travel attitude, environmentally careless holiday attitude (i.e., not caring about the environment when planning vacation), perceived behavioral control, and efficacy beliefs that reducing aviation contributes to climate change mitigation.

Individual behavior change can only make a difference on a large scale if many people (especially from affluent countries) alter consumer choices. Therefore, the collective dimension of environmentally relevant behavior has received greater attention in recent years. Particularly, it has been argued that people’s identification with relevant social groups might drive pro-environmental behavior [see e.g., Ferguson et al. (2016) and Fritsche et al. (2018)]. Social Identity Theory and Self-Categorization Theory state that people do not only define themselves as individuals via different aspects of their personal identity but also as members belonging to different social groups (Tajfel and Turner, 1986; Turner et al., 1987). Moreover, people can also identify on a global level with all humanity. Some authors have thus examined the relevance of a global identity (i.e., perceiving a group membership with all humanity, McFarland et al., 2012, 2019), for counteracting environmental crises (e.g., Running, 2013; Der-Karabetian et al., 2014; Barth et al., 2015; Joanes, 2019; Loy and Reese, 2019; Loy and Spence, 2020; Loy et al., 2022a). Loy et al. (2022b), for example, argued that global identity could serve as driver to act in collective instead of self-interests when it comes to climate change mitigation. They found that identification with people all over the world predicted self-reported climate-protective behavior and behavioral intentions. Römpke et al. (2019) argued that intergroup contact could promote collective action to solve global crises *via* strengthening global identity. The authors found that international contact increased global identity and also diverse pro-environmental and social behaviors.

Global identity might be particularly relevant when it comes to air travel. On the one hand, global identity might drive people’s interest in long-distance journeys and thereby foster air traveling. On the other hand, it might prevent people from this highly CO_2_-intensive behavior threatening their global ingroup [see (Loy et al., 2021)]. Previous research revealed mixed evidence so far. Römpke et al. (2019) found no relation between global identity and the intention to avoid air travel. Loy et al. (2021) found a positive relation between global identity and self-reported previous refrain from air travel as well as policy support to decarbonize the mobility system. Global identity was not associated with how frequently people had stayed abroad and negatively with CO_2_ emissions calculated from self-reported previous flights. However, global identity was positively related to the quantity and quality of contact with locals during traveling and situationally increased when participants were asked to think of past international travel experiences. Therefore, Loy et al. (2021) concluded that global identity seems to be no hindrance for decarbonizing mobility, including the willingness to fly less, even though in-depth international contact seems to be advantageous. In our study, we thus included global identity as further possible predictor of people’s intention to refrain from flying.

#### Psychological Determinants of Policy Support

In addition to personal mobility behaviors, approval for policies that regulate air traffic is also important for decarbonizing transport. Past literature identified several factors that influence approval of environmental policies, such as the perceived fairness and effectiveness of policies, risk perception, concern and knowledge about environmental problems, trust, values, political orientation, or social norms (Kallbekken and Sælen, 2011; Drews and van den Bergh, 2016; Fairbrother et al., 2019; Bergquist et al., 2022). However, most of the conducted studies dealing with policy support in the transport sector focus on everyday behavior (i.e., reduction of car usage, diffusion of electric vehicles or modal shift; Schade and Schlag, 2003; Pridmore and Miola, 2011; Schuitema et al., 2011; Schmitz et al., 2019). Only few studies have specifically focused on support for environmental policies regarding long-distance travel, and in particular, aviation [see e.g., Kallbekken and Sælen (2021)]. Kallbekken and Sælen (2011) found that next to many of the above-named variables, perceived effectiveness, perceived threat and imminence of problems, knowledge, trust, and expected negative effects of policy measures on the self and the poor explained policy support.

As argued for intentions to reduce air travel, also respective policies might be favored by globally identified people. Accordingly, Loy and Reese (2019) found that the stronger people’s global identity, the stronger they supported climate policies [see also Loy et al. (2022a)]. More specifically, Loy et al. (2021) found a positive correlation between global identity and support of policies to decarbonize the mobility system. In our study, we thus included global identity as further possible predictor of people’s policy support.

Moreover, the perceived fairness of policies is a particularly strong correlate of policy acceptance (Bergquist et al., 2022). Fairness perception might depend on individual justice concerns as well as the salience of inequalities. Therefore, in the following, we discuss the relation between air travel and different inequalities and related justice concerns, and how these concepts were integrated into our study.

### Air Travel and Its Relation to Different Inequalities

Even though the terms inequality and injustice are related to each other and both used in this article, they are not equivalent (Hegtvedt and Isom, 2014). Inequality refers to a rather objective circumstance that can be determined by judging parameters (e.g., income). Injustice, in turn, implies a subjective assessment of these circumstances. What is perceived as just and unjust differs between individuals (Hegtvedt and Isom, 2014), for example, depending on how justified they consider inequalities between people (Reese et al., 2014). The perception of (in)justice and moral reasoning are relevant aspects of psychological functioning and explain how people behave (Killen and Dahl, 2021). People may (re-)act differently to perceived moral norm violations and injustice according to their individual definition of (in)justice and a general sensitivity for (in)justice (Dar and Resh, 2001). In order to understand people’s behavior, it is therefore crucial to identify people’s moral and justice ideals and concerns (Rothmund et al., 2016).

A growing body of research has linked people’s justice perceptions to environmental issues (Clayton et al., 2016; Jenkins et al., 2016; van der Horst et al., 2021; Zabern and Tulloch, 2021). In debates on energy transition, several inequality issues have been pointed out [see e.g., Jenkins et al. (2016)]. The distribution of resources and costs (Meyer, 2019) but also energy consumption and resulting emissions (Pellegrini-Masini et al., 2020) entail relevant (distributional) inequalities. For example, Oswald et al. (2020) report that among 86 countries, the top 5% consume more than 20% of the final energy use. This inequality in energy consumption, however, does not translate into a corresponding burden sharing of the (negative) impacts. Even though climate change threats are expected to affect all people, vulnerable groups, particularly in the Global South, are most strongly endangered (Parks and Roberts, 2006).

When the perception of injustice leads to feelings of anger, it is considered to initiate action against this injustice, including collective action (Landmann and Rohmann, 2020). In our research, we are interested in distributional justice regarding the accessibility and (negative) impact of air travel. In the following, we outline how air travel is related to different distributional inequalities and how perceiving them as injustices might be related to people’s intentions to refrain from flying and support policies to reduce air travel.

#### Global Inequality

Global inequality is often associated with inequalities between people all over the world regarding economic parameters such as income and purchasing power (Milanovic, 2011) or poverty (Pieterse, 2002). Global inequality in terms of climate change refers to the unequal distribution of climate change effects (i.e., effects are stronger in the Global South compared to the Global North) as well as to the proportionate contribution causing the climate crisis [i.e., contribution is smaller in the Global South than in the Global North; IPCC (2022b)]. Climate change effects, in turn, will intensify economic inequalities (IPCC, 2022a). Several global injustice issues are debated regarding the aviation sector. For example, only a small part of the world population flies on a regular basis. Gössling and Humpe (2020) found that 1% of the world population is responsible for 50% of the CO_2_ emissions resulting from commercial aviation.

Reese et al. (2014) showed that people who perceived global inequalities as injustice were more likely to intend actions in favor of global equality. Environmentally concerned and knowing people are generally aware of global injustices related to climate change (Waldron et al., 2019). At the same time, environmental concern (alone) is not necessarily associated with abstaining from air travel (Alcock et al., 2017). Still, people who considered global justice of resource use important reported stronger intentions to act pro-environmentally (Reese and Jacob, 2015). Reese et al. (2014) argued that globally identified people should particularly perceive global inequalities as unjust. Accordingly, they found that the stronger people’s global identity, the stronger their perception of global injustice, which in turn predicted their intentions to act in favor of global equality. Based on these results, Reese (2016) further argued that global identity might strengthen perceptions of global inequalities with regard to climate change effects and thereby motivate transnational efforts to mitigate climate change.

Inferred from this reasoning, we examined whether making global inequalities related to air travel salient influences people’s intentions and policy support to reduce air travel and whether this depends on people’s global identity.

#### Intranational Inequality

Inequality occurs not only at the global level but also at the intranational level. Many researchers examined economic and educational inequalities between citizens of a nation (Hoover, 1989; Odoardi et al., 2020). Moreover, an increasing number of studies outlined unequal climate change causation and distribution of climate change impacts at the national level (Rao, 2014; Oswald et al., 2020). Both considerations can also be applied to the context of air travel behavior. The aviation sector is a highly subsidized sector with several levels of government providing financial assistance to support airports, aircraft, and airlines. The cheap tickets made possible by this do not reflect the true costs of air travel (even without accounting for environmental externalities; Transport and Environment, 2019). Among various subsidies at regional, national, and EU levels, tax exemptions on fuel and tickets are often considered the largest subsidy (Transport and Environment, 2019; Gössling and Humpe, 2020). In addition, due to the COVID-19 pandemic and the subsequent decline in passenger travel, many countries have granted airlines great amounts of subsidies (e.g., loans, credit guarantees, and state aid) with mostly no climate or dividend conditions (Transport and Environment, 2021).

From an intranational equality perspective, the question arises what share of the population benefits from these subsidies that are enabled by all taxpayers (and accordingly emits greenhouse gas emissions). On a national level, the amount of people who don’t fly (on an annual basis) outnumber those who do. Surveys indicate that the most frequent flyers (between 3.7 and 12% of the flying population depending on country and statistic) accounted for 28.8–68% of all flights [for more details, see Gössling and Humpe (2020)]. In addition, these frequent flyers who benefit the most from subsidized air travel tend to belong to the wealthy part of the population. From a distributional justice perspective, subsidies for air traffic thus raise allocational questions (Gössling and Humpe, 2020).

Similar to the motivating potential of global identity to reduce global injustice, Reese (2016) argued that a regional identity (e.g., national identity) might strengthen perceptions of regional injustices with regard to climate change effects and thereby motivate regional efforts to mitigate climate change.

Making the intranational inequalities (i.e., the subsidies) of air travel salient could thus encourage intentions to avoid flights and lead to higher support for policies that regulate air travel. These effects might depend on people’s national identity.

#### Intergenerational Inequality

The duty of current generations to preserve the livelihood of future generations has been discussed from the perspective of various disciplines, especially philosophy and economics (Rawls, 1971; Tobin, 1974; Ball, 2001; McCormick, 2009; Hay, 2015). The imbalance of causing and carrying environmental problems between generations (Zabern and Tulloch, 2021) is referred to as intergenerational inequality, as the costs on ecosystems and humans will to a large extent be carried by younger and future generations (Hansen et al., 2013). In political debates, the necessity of viewing the impacts of certain policies from an intergenerational perspective is considered to be crucial in order to provide a sustainable societal and ecological system for the future (Buchanan, 2020). How this discrepancy is perceived and appraised will in the following be referred to as concern for intergenerational justice.

In the case of the climate crisis, the link between intergenerational injustice and climate change has already been made decades ago (Page, 1999). The notion of intergenerational injustice addresses questions regarding the distribution of social, economic, and cultural resources amongst present and future generations as climate change will impact the life of future generations adversely (Page, 1999; Skillington, 2019; IPCC, 2022a). Increasing air traffic and its impact on the climate contribute to the (possible) inability to keep the global temperature increase below 1.5°C compared to pre-industrial levels (Scott et al., 2015). With the rise of youth activists like Greta Thunberg and the Fridays for Future movement (Leung, 2020), the concept of intergenerational justice has gained increasing attention. Climate action was framed as an issue of intergenerational justice effort, aiming to preserve ecosystems for future generations (Zabern and Tulloch, 2021).

Gössling et al. (2020) provided examples of headlines from newspaper articles. They revealed that the relation of air travel with intergenerational injustice is perceived and communicated as moral concern. Reese and Jacob (2015) found that the more people valued intergenerational justice of resource use, the stronger their intentions to act pro-environmentally. This relation was mediated by feelings of moral anger and responsibility. Similarly, Syme et al. (2000) found that perceived intergenerational injustice motivates people to engage in pro-environmental behavior and support policy measures for protecting the environment.

Building on these findings, we examined whether intergenerational justice concerns are positively related to the intention to avoid air travel and the support for regulative policies to reduce air travel.

### Aim and Hypotheses of the Study

The present study seeks to identify psychological factors that are associated with the intention to avoid flights and policy support to reduce air travel. The focus of this study is air travel behavior within Europe which is more or less easily replaceable by other transport means. We hypothesized that pro-travel and environmentally careless holiday attitudes are negatively related to intentions to refrain from air travel, while perceived behavioral control to travel without flying, efficacy beliefs, global identity, and concern for intergenerational justice are positively related to intentions to refrain from air travel. With regard to policy support to reduce air travel, we assumed that global identity and concern for intergenerational justice are positively associated. Since intentions to avoid flights and policy support to reduce aviation are both expressions of pro-environmentalism [the former in the private sphere, the latter in the public sphere, see Stern (2000)], we also examined the variables that were not explicitly hypothesized as predictors of both.

In addition, we hypothesized that information on air travel-related global and intranational inequalities will encourage the intention to avoid flights and policy support to reduce air travel—depending on people’s identity. Specifically, we expected that globally identified people might respond more strongly to information regarding the global injustice of air travel, while nationally identified people might respond more strongly to information regarding intranational inequalities induced by subsidies. To control for differences in knowledge, we included subjective knowledge regarding air travel’s impact on climate change as control variable in the analyses. Moreover, we examined the role of income and education as well as further demographic characteristics (age, gender).

## Materials and Methods

The study was conducted in October 2019 as part of a bigger research project (“Sozio-E2S”) assessing mobility and investment behavior for energy system modeling.

### Study Design

**FIGURE 1:**
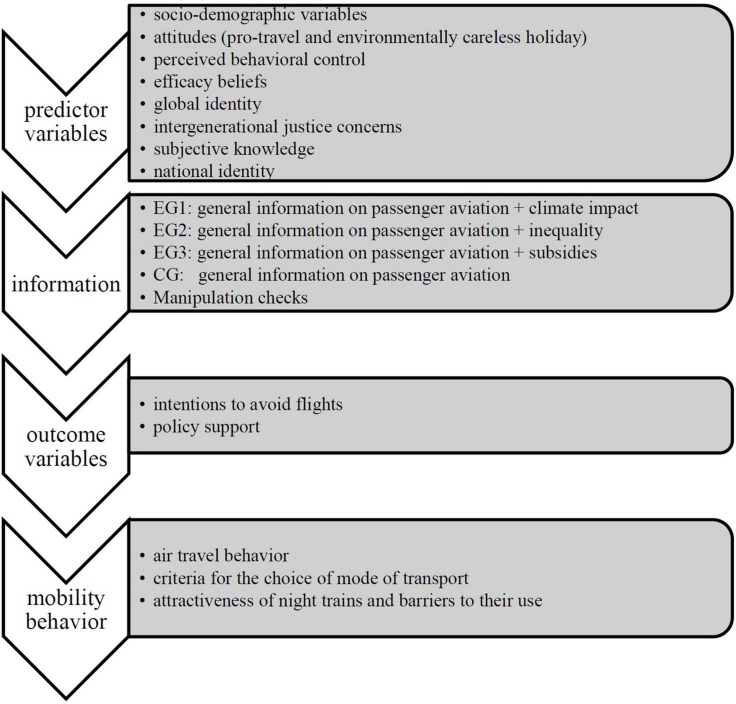
Survey flow.

In order to examine the relationships between the hypothesized psychological predictors and the two outcome variables (intentions and policy support to reduce air travel), we employed a questionnaire comprising all relevant variables and conducted regression analyses. We included a between-subjects experimental factor to analyze the effect of informing respondents about different inequalities related to air travel on intentions and policy support to reduce air travel. Participants were randomly assigned to one of four groups (three experimental conditions and one control condition). All groups received the same introductory information about the growth of air traffic. In addition, Experimental Group 1 received information about the environmental/climate effects of aviation. This group was added in order to compare inequality information to factual information on climate impact. Experimental Group 2 received information about aspects of global and intranational inequality of aviation, Experimental Group 3 about German subsidies for the aviation sector implying intranational inequality. The text information can be found in Table 1. The summary of the survey flow is depicted in Figure 1.

**TABLE 1:** Information provided in the three experimental conditions and the control condition.

**General information on passenger aviation (introduction for all conditions)** Air traffic is growing rapidly both globally and at European and national level. Since 1990, the annual number of passengers has increased by 100% globally—in Germany by as much as 243%. Within the next two decades, the International Air Transport Association expects a further doubling of air transport.

**1. Experimental condition 1: Environmental effects** This makes aviation the industry with the fastest growing emissions. In addition to CO_2_, other substances are released during a flight, which have varying degrees of warming or cooling effects. In total, however, they increase the climate impact of flying. Depending on the source, it ranges between a factor of 2 and a factor of 4 of the direct CO_2_ equivalents (“Radiative Forcing Index RFI”). In 2017, air traffic in and from Germany generated about 31 million tons of CO_2_ equivalents. If the non-CO_2_ effects are included in the estimate (factor 3, see RFI), the climate impact of German air traffic would correspond to emissions of approximately 94 million tons of CO_2_ equivalents. This means that the percentage of German aviation in the overall climate impact is around 9.7%.

**2. Experimental condition 2: Aviation and justice** Emissions from air transport are unfairly distributed both globally and within Germany: it is estimated that less than 5% of the world’s population has ever been on an airplane. Latin America and Africa account for only 11% of air traffic, while North America and Europe, with lower total populations, account for more than half. Overall, only a minority of Germans fly regularly. A recent survey by Infratest Dimap found that six out of ten people in Germany rarely or never fly at all. People in the highest income group in Germany fly an average of 6.6 times per year, while those in the lowest income group fly 0.6 times per year.

**3. Experimental condition 3: Aviation and subsidies** The aviation sector in Germany is massively subsidized by the general public. Both indirect and direct subsidies are listed below: 1) Tax on kerosene By exempting kerosene from energy tax, the state waived the industry around seven billion euros in tax revenues every year. 2) Value-added tax/sales tax There is no VAT on international airline tickets, which means that the state loses around five billion euros in tax revenue. 3) Subsidies for airports in Germany 10 of the 16 international airports in Germany are in the red and permanently dependent on public subsidies. Of the 19 regional airports, not a single one is self-sustaining. As a rule, the annual loss is over 100 million euros. In addition, domestic air traffic alone caused external environmental costs (i.e., the health, environmental and climate damage to be borne by the general public) of 1.3 billion euros in 2017.

**4. No additional information**

To check whether information was read and understood, we included manipulation checks after the information was provided (1. “Aviation accounts for a large share of the total climate impact of German greenhouse gas emissions”, 2. “Emissions from aviation are (globally) unfairly distributed”, 3. “(German) air traffic is heavily subsidized” answered on a 5-point Likert Scale from 1 (*strongly disagree*) to 5 (*strongly agree*). Manipulation checks were successful except for the climate impact of aviation. No differences were found between the groups for this item. We assume that either the information text was not distinct enough in stressing the climate impact in contrast to the other groups, that participants were already similarly aware about the climate impact, or that the manipulation check was too vaguely formulated.

The survey was conducted online. Programming and data collection was done by the market research institute Aproxima GmbH. The questionnaire was sent to a quota sample of 2,800 citizens mirroring the population in relevant sociodemographic characteristics. An evaluation of the market research institute showed that the majority of respondents were very or rather satisfied with the questionnaire, which suggests a positive effect on response quality.

### Sample

#### Sampling Procedure and Socio-Demographic Characteristics

Participants were recruited with quotas on age, gender, education, income, city size, and geographical region to ensure a distribution mirroring the German population (above the age of 16) in these characteristics (see Table 2). In total, 2,530 respondents completed the survey, from which 20 were omitted due to repetitive answer patterns. Respondents received incentives to participate from the provider of the online access panel. Data control for anomalies was carried out by the market research agency after the first 50 cases as well as in the evaluation phase.

**TABLE 2:** Socio-demographic characteristics of sample and population.

Variable	Classification	Population (in %)	Weighted sample (in %)
Gender	Male	49	48.6
	Female	51	51.1
	Diverse		0.2
Age	16–25	12.5	12.2
	26–35	15.1	15.1
	36–49	20.9	20.6
	50–65	27.9	28.3
	66+	23.6	23.6
Formal education	Still in school	3.3	3.5
	None	3.7	2.2
	Low	30.4	30.2
	Medium	30.2	31.1
	High^a^	32.4	32.8
Income^b^	Less than 1.000€	9.6	12.2
	1.000–1.999€	26.1	27.7
	2.000–2.999€	23.5	27.7
	3.000–3.999€	16.1	16.6
	4.000–4.999€	10.3	9.5
	5.000€ or more	13.6	5.4
City size	Less than 5.000	14.3	14.8
	5.000–19.999	26.5	25.9
	20.000–99.999	27.4	27.5
	100.000 and more	31.8	31.4

*Geographical region was representatively distributed in accordance with German states. ^a^High formal education is referred to here as university entrance qualification (Abitur, including “Fachhochschulreife”). The population indicators were provided by the market research institute Aproxima with the exception of income. ^b^For the income distribution among the German population, data from the Federal Statistical Office (Statistisches Bundesamt, 2020) were used.*

Due to the quota system, the unweighted sample was close to the population in almost all parameters. Subsequent weighting was carried out primarily for the school-leaving qualifications “without a school-leaving qualification” and “university entrance qualification” and for the location size categories “under 5,000 inhabitants” and “5,000 to under 20,000 inhabitants.” In the weighted sample, all parameters considered were less than 1% point different from the population. For conducting regression analyses we dummy-coded levels of formal education and gender. Higher education was coded as 1 (*university entrance certificate including “Fachhochschulreife” to university degree*) and 0 [*lower education*, see also Dütschke et al. (2022)]. In our sample 32.8% of the respondents held a higher education, thus mirroring the German population closely (32.4%). Gender was coded as 1 (*female, divers*) and 0 (*male*).

#### Air Travel Behavior and Modes of Transport

Of all participants, 84.7% indicated to have taken a plane before, 15% had never taken one, and 0.2% did not specify. Most participants had not taken a plane within the past 12 months at all within Europe. In the case of travel due to leisure/vacation reasons within Europe, two thirds of the respondents had not taken a plane at all (66.32%) and less than 10% had taken more than one return flight (1 single flight = 7.93%, 2 single flights = 17.58%, 3 single flights = 0.89%, 4 or more single flights = 7.28%; the maximum number of single flights in this group was 30, followed by 18 and 16 single flights). Younger and highly educated respondents in our sample tended to fly more than older and less educated respondents as illustrated in Table 3.

**TABLE 3:** Average amount of flights differentiated by age and formal education.

Variable	Classification	Mean (number of flights for vacation in the last year)
Age	16–25	1.93
	26–35	1.65
	36–49	1.33
	50–65	1.27
	66+	1.02
Formal education	Still in school	1.89
	None	0.48
	High school diploma (9 years of school)	1.02
	University entrance certificate	1.68
	University degree	1.93

*Extreme values >3 SD were removed to minimize the impact of very frequent flyers on the statistics. As stated before, more than two thirds of the respondents indicated to not have taken a plane at all within Europe within the past year for vacation reasons.*

When asked about their preferred mode of transport for distances between 500 and 1,000 km, 50.5% of the participants reported to choose a private car, 27.6% a train, 13.2% a plane, 4.7% a public bus. Car sharing, car rental, and company cars were each preferred by less than 1% of the respondents.

We also asked participants to rank criteria that influence which mode of transport they choose for leisure activities. The criteria which were most important to our sample are travel time and price. Environmental friendliness was assigned a rather low importance by the respondents, with 41.6% naming it to be the least important criterion. To 6.4% of the respondents, it was the most important one. Table 4 provides details about the importance of the criteria for means of transport.

**TABLE 4:** Criteria for choice of transport means between 100 and 500 km.

Criterion	Rank 1	Rank 2	Rank 3	Rank 4	Rank 5
Travel time	31.4%*	31.2%	22.1%	10.6%	4.6%
Price	33.0%	29.2%	19.4%	13.0%	5.3%
Comfort	16.0%	22.4%	28.9%	20.8%	11.9%
Habit	18.5%	10.5%	14.5%	23.7%	32.9%
Environmental friendliness	6.4%	8.2%	14.8%	29.0%	41.6%

“What criteria do you use to decide which means of transport you use for your leisure activities for distances between 100 and 500 km? Please rank them accordingly.” Rank 1 = most important criterion, rank 5 = lowest importance. *Example: 31.4% of the participants ranked travel time as the most important criterion for choosing their means of transport.

Since night trains are a possible alternative option especially for trips to other European countries, we additionally assessed past use and attractiveness of night trains. For the majority of respondents, night trains were attractive both within Germany and within Europe (*M*_*Europe*_ = 3.63, *SD* = 1.24; *M*_*Germany*_ = 3.5, *SD* = 1.3; scale ranging from 1 = *not attractive at all* to 5 = *very attractive*). However, 65% of respondents had never traveled on a night train. For 28%, it had “been a while” and only 7% had traveled by night train in the past two years. Several issues discourage our sample from using night trains more often. They named a lack of information (76%), that trains travel too infrequently (80%), are too expensive (27%), that no suitable connections are available for relevant destinations (22%), that night trains are not needed (26%), that they had not yet considered to travel by night trains (35%), that travel times of night trains are too long (13%), night trains are uncomfortable (13%), or other reasons (8.4%). Furthermore, 62% of the respondents rather or fully agreed that more night trains should be used within Europe, 10% rather or fully disagreed (*M* = 3.8, *SD* = 1.09; scale ranging from 1 = *don’t agree at all* to 5 = *fully agree*).

### Measures

Participants answered all items on 5-point Likert scales ranging from 1 (*don’t agree at all*) to 5 (*fully agree*). A comprehensive item list with descriptions can be found in Supplementary Table 1.

#### Outcome Variables

*Intention to avoid flights* was measured with one item (“In the future, I will try to avoid flights in general”), following the concept of behavioral intention from the TPB (Ajzen, 1991).

*Policy support* was measured with eight items (e.g. “Overall, I am in favor of policies being put in place to reduce air traffic as a whole.”, α = 0.91) following media discourses (e.g., Stay Grounded, 2022) and previous studies examining multiple policies in the transport sector (Schade and Schlag, 2003; Eriksson et al., 2008).

#### Predictor Variables

*Pro-travel attitude* was measured with three items from Barr and Prillwitz (2012; e.g., “All people in Germany should have the opportunity to vacation wherever they want in the world”). Due to poor internal consistency, only the named item was used for the analyses. *Environmentally careless holiday attitude* was adapted from pro-environmental holiday attitude introduced by Barr and Prillwitz (2012), yet due to content reasons measured with only one item that we refer to as environmentally careless holiday attitude (“I don’t worry about the environment when I make decisions about my vacation travel”). *Perceived behavioral control* [adapted from TPB, Ajzen (1991); “I have enough options for a good vacation without having to fly”] and *efficacy beliefs* [following the operationalization of self-efficacy beliefs from Doran et al. (2015), and considering outcome expectancy from Maddux et al. (1982); “Avoiding air travel helps fighting climate change”] were assessed with one item each.

*Global identity* was measured with two items (e.g., “Overall, being a citizen of the world is an important part of how I see myself”, *r* = 0.51^**^) selected from Buchan et al. (2011). *National identity* was adapted from Lilli and Diehl (1999), with only two (*r* = 0.55^**^) out of three items used for the further analyses due to poor internal consistency (e.g., “The nation I belong to is an important reflection of who I am”).

Items for assessing *intergenerational justice concerns* were built upon theoretical work from Russell et al. (2003) but shortened to five items and adjusted to the context (e.g., “We should take up all efforts to secure the livelihood of future generations”). Due to poor internal consistency, two items were excluded from further analyses (α = 0.81).

Finally, we assessed *subjective knowledge* with one item [“I could spontaneously explain to a friend what air travel has to do with climate change”; Davison et al. (2014)].

### Data Analysis

Data was analyzed using IBM SPSS (version 20). Oneway ANOVAs were conducted to test whether the provision of different textual information regarding air travel had an effect on intentions to avoid flights or on policy support. Further, we calculated bivariate correlations (Pearson coefficient), multiple linear regression analyses with step-wise uptake of variables including age, gender, income, education, and subjective knowledge as control variables. In a first step, the predictor variables as well as dummy-coded comparisons between experimental conditions and other experimental conditions as well as control condition were included in the regression model. In a second step, we added the interaction terms with global and national identity.

## Results

### Bivariate Correlations Between Variables

As displayed in Table 5, there was a strong positive correlation between the two outcome variables intention to avoid flights and policy support. Efficacy beliefs, perceived behavioral control to travel without flying, and intergenerational justice concerns revealed moderate or strong positive correlations with the outcome variables. Pro-travel attitude and environmentally careless holiday attitude had moderate negative correlations, global identity had weak positive correlations with both. Among the further assessed (control) variables, age, self-estimated knowledge, and national identity showed weak positive relationships with intentions to avoid flights and policy support. The correlations between the explanatory variables were weak to moderate except for intergenerational justice concern. The latter correlated moderately with efficacy beliefs and global identity.

**TABLE 5:** Bivariate correlations of the predictor variables and outcome measures addressed in this study.

Variable	1	2	3	4	5	6	7	8	9	10	11
1 Intentions to avoid flights	1										
2 Policy support	0.59***	1									
3 Age	0.16***	0.09***	1								
4 Income	–0.04	–0.03	–0.02	1							
5 Pro-travel attitude	–0.30***	–0.32***	–0.07***	0.02	1						
6 Environmentally careless holiday attitude	–0.26***	–0.31***	–0.00	0.06**	0.25***	1					
7 Perceived behavioral control	0.51***	0.38***	0.07**	–0.03	–0.15***	–0.16***	1				
8 Efficacy beliefs	0.46***	0.53***	0.00	–0.02	–0.18***	–0.22***	0.33***	1			
9 Global identity	0.07**	0.17***	0.09***	–0.04	0.02	–0.07**	0.06**	0.16***	1		
10 Intergenerational justice concerns	0.37***	0.50***	0.03	–0.02	–0.16***	–0.28***	0.28***	0.46***	0.41***	1	
11 Subjective knowledge	0.16***	0.28***	–0.06**	0.00	–0.09***	–0.11**	0.15***	0.32***	0.22***	0.31***	1
12 National identity	0.08***	0.08***	0.21***	0.01	0.11***	0.06**	0.09***	0.06**	22***	0.16***	0.04*

*p < 0.05, **p < 0.01, ***p < 0.001.

### Predictors of Intentions and Policy Support to Reduce Flights

In a next step, we conducted a multiple linear regression analysis to examine the strength of the relations between the assumed predictor variables and people’s intention to avoid flights, controlling for the other predictors as well as the control variables (see Table 6). The model explained 43% of the variance in people’s intention to avoid flights. The hypotheses that pro-travel attitude and environmentally careless holiday attitude negatively predict intentions were supported. Further, in line with our hypotheses, perceived behavioral control to travel without flying, efficacy beliefs that avoiding flights helps to mitigate climate change, and concern for intergenerational justice positively predicted intentions to refrain from air travel. Perceived behavioral control was the strongest predictor: particularly participants indicating to have sufficient options for traveling without planes had higher intentions to avoid travel. The regression further revealed a small but significant negative relationship between global identity and intentions to avoid flights, contrasting the hypothesized positive relation that was found in the bivariate analysis. Global identity and concern for intergenerational justice were positively related in our study. As justice concerns explained variance in people’s intentions to avoid flights, there might be a suppressor effect. In support of this, the negative relation between global identity and intentions vanished (β = -0.01, *p* = 0.489), when we removed intergenerational justice concerns from the model.

**TABLE 6:** Results of linear multiple regression of intentions to avoid flights.

Predictor	*B*	SE	β	*t*
Age	0.01	0.00	0.11	7.09***
High education (dummy)	–0.35	0.05	–0.11	–6.80***
Pro-travel attitude	–0.20	0.02	–0.17	–9.96***
Environmentally careless holiday attitude	–0.08	0.02	–0.07	–4.20***
Perceived behavioral control	0.43	0.02	0.35	20.30***
Efficacy beliefs	0.30	0.02	0.26	13.59***
Global identity	–0.08	0.03	–0.05	–2.81**
Intergenerational justice concerns	0.30	0.04	0.14	6.84***

Final model. Income, gender, and subjective knowledge were not significant predictors and excluded from the final model. Furthermore, neither information on climate impact (dummy, other experimental groups and control group = 0), information on (global) inequality (dummy, other experimental groups and control group = 0), nor information on subsidies (dummy, other experimental groups and control group = 0) significantly predicted intentions to avoid travel. R^2^_corr_ = 0.43 (p < 0.01). **p < 0.01, ***p < 0.001.

Among the control variables, age was positively, and education negatively related to intentions to avoid flights. Income, gender, and subjective knowledge about the impact of air travel for climate change showed no significant association with people’s intention to avoid flights.

The second regression model explained 45% of the variance in people’s support of policies to reduce air travel (see Table 7). Our hypothesis that concern for intergenerational justice is positively related was confirmed. However, contrary to our hypothesis, global identity was not a significant predictor of policy support. Yet, global identity became a positive predictor of policy support (β = 0.08, *p* < 0.001), when we removed intergenerational justice concerns from the model. Among the control variables, age was positively, and high education negatively related to policy support. Furthermore, perceived behavioral control to travel without flying, efficacy beliefs, and subjective knowledge revealed significant positive relationships with policy support, while environmentally careless holiday attitude and pro-travel attitude were negative predictors. Overall, efficacy beliefs and concern for intergenerational justice were the strongest predictors of people’s support for restricting flights by political measures. Income and gender were no significant predictors.

**TABLE 7:** Results of linear multiple regression of policy support to reduce flights.

Predictor	*B*	SE	β	*t*
Age	0.00	0.00	0.06	3.78***
High education (dummy)	–0.08	0.03	–0.04	–2.47*
Pro-travel attitude	–0.15	0.01	–0.17	–10.23***
Environmentally careless holiday attitude	–0.07	0.01	–0.09	–5.36***
Perceived behavioral control	0.14	0.01	0.17	9.81***
Efficacy beliefs	0.22	0.02	0.29	15.36***
Intergenerational justice concerns	0.37	0.03	0.26	12.92***
Subjective knowledge	0.05	0.01	0.07	3.73***

Final model. Income, gender, and global identity were not significant predictors and excluded from the final model. Furthermore, neither information on climate impact (dummy, other experimental groups and control group = 0), information on (global) inequality (dummy, other experimental groups and control group = 0), nor information on subsidies (dummy, other experimental groups and control group = 0) significantly predicted policy support. R^2^_corr_ = 0.45 (p <.001). *p < 0.05, ***p < 0.001.

### Effect of Information on Intentions and Policy Support to Reduce Flights

Comparing the three experimental groups and the control group with oneway ANOVAs revealed that the provision of different textual information regarding air travel had neither an effect on intentions to avoid flights (*p* = 0.396) nor on policy support (*p* = 0.296). Adding three dummy variables for the information conditions (compared to the three other conditions, respectively) in the regression analyses also showed no effects (Tables 6, 7). Interaction terms between information and identity were added in a second step. Disconfirming our hypotheses, neither the interaction between information on (global) inequalities and global identity, nor the interaction between information on subsidies (implying intranational inequalities) and national identity were significant (see Supplementary Tables 2, 3 for details). Hence, more globally identified people were not more responsive to information including global inequalities, more nationally identified people were not more responsive to information on subsidies implying intranational inequalities. In the regression analysis examining intentions to avoid flights including interaction terms, the experimental condition comprising information on climate impact turned significant (*p* = 0.044), yet with a very small effect size (β = -0.04). As the ANOVA as well as the regression analysis containing the experimental conditions without interaction terms revealed no significant effect of either information condition, we will refrain from further interpreting this (minor) effect.

## Discussion

### Summary of the Results and Theoretical Contribution

With this study, we investigated the relationship between pro-travel attitude, environmentally careless holiday attitude, perceived behavioral control to travel without flying, efficacy beliefs, global identity, and concern for intergenerational justice with both, the intentions to avoid air travel as well as policy support to reduce aviation. Moreover, we hypothesized that information on global and intranational inequalities related to air travel will encourage the intention to avoid flights and policy support to reduce air travel—depending on people’s identity. Specifically, we expected that globally identified people might respond more strongly to information regarding the global injustice of air travel, while nationally identified people might respond more strongly to information regarding the intranational inequalities induced by subsidies.

#### Predictors of Intentions to Avoid Flights

In our sample, we found that perceived behavioral control over (sustainable) travel modes, the efficacy belief that avoiding flights helps to mitigate climate change, lower pro-travel attitude, intergenerational justice concerns, age, lower education, lower environmentally careless holiday attitude, and lower global identity predicted intentions to avoid flying in descending order (in terms of strength of the relationship). Subjective knowledge was the only factor not significantly associated with intentions to avoid flying.

The finding that perceived behavioral control to travel without flying was the strongest predictor for intentions to avoid flights is in line with previous research (Dütschke et al., 2022) and in accordance with the TPB that considers perceived behavioral control as a relevant factor explaining planned behavior in general, and pro-environmental behavior in specific (Yuriev et al., 2020). Our results highlight the importance of this factor for pro-environmental behavioral intentions in the mobility sector. Efficacy belief was the second strongest predictor of intentions to avoid flights. Believing that personal behavior makes a difference for the desired outcome (in our case climate change mitigation) thus constitutes another important factor for mobility choices resonating with previous research regarding antecedents of pro-environmental behavior (Doran et al., 2015; Jugert et al., 2016).

Furthermore, the concern for intergenerational justice was positively related to intentions to avoid flights. So far, only few psychological research has dealt with concerns for intergenerational justice. For example, Reese and Jacob (2015) found intergenerational justice to predict pro-environmental intentions independently but indirectly via responsibility and anger. Hence, our findings support and transfer previous results to the concrete topic of flight reduction.

Intentions to avoid flights were higher for participants who consider the environmental impact when planning vacation (negative relation with environmentally careless holiday attitude). People who were convinced that everyone should be able to go on vacation wherever they want showed lower intentions to avoid flights (pro-travel attitude). These findings resonate with prior research (Barr and Prillwitz, 2012). People with such travel attitudes thus might weigh (personal) freedom aspects higher than the environmental impact of traveling.

Furthermore, age was positively related to intentions to avoid flights. Prior research is inconsistent with regard to the relation between age and pro-environmental behaviors (e.g., Wiernik et al., 2013; Otto and Kaiser, 2014). However, our results suggest that avoiding flights is especially unattractive or difficult for younger people who might still have a stronger desire to explore the world. Our results of a negative relation between education and intentions to avoid flights contrast with previous literature showing that the level of education was positively associated with intentions to refrain from air travel (Dütschke et al., 2022). However, the sample of Dütschke et al. (2022) differed from our sample in terms of share of respondents with a university degree. Their sample indicated a higher formal educational status than ours which might explain differences in results. Furthermore, contrary to Reichert and Holz-Rau (2015) but in line with Dütschke et al. (2022), income was not a significant predictor of intentions to avoid flights in our study.

Despite a small positive bivariate correlation with intentions to reduce air travel, global identity negatively predicted intentions to avoid flights in the regression analysis accounting for all other predictors—with a very small effect size. Comparing this result with prior evidence reveals an inconsistent picture. While Römpke et al. (2019) found no association between global identity and personal intentions to reduce flying, Loy et al. (2021) found that people scoring higher on global identity stated to have refrained from air travel more often than less globally identified persons (but this relation vanished when controlling for people’s sufficiency orientation). Characteristics of the different samples might be one reason for differing results. Loy et al. (2021) had a sample that was younger and educated above average, socio-demographic characteristics that come along with higher numbers of flights [Kreil (2021); see also our results]. At the same time, they might be more aware about the negative side effects than the general population. A conflict of interests might arise for highly globally identified individuals. International personal contact, even fictitious one, can strengthen global identification (Römpke et al., 2019). Reversely, such contact might be more important to and aspired by individuals who identify strongly with the world community. Accordingly, Sparkman and Eidelman (2018) found that the number of travels outside the US was positively related with US citizens’ global identity. Getting to know other cultures and exploring the world hence potentially constitutes a valuable benefit for these individuals outweighing environmental concerns. Another explanation why global identity was a negative predictor of intentions in the regression analysis (despite a positive bivariate relation) might be that global identity and concern for intergenerational justice were positively related in our study, in line with previous findings (see McFarland et al., 2019). As justice concerns explained variance in people’s intentions to avoid flights, there might be a suppressor effect. In support of this, the negative relation between global identity and intentions vanished, when we removed intergenerational justice concerns from the model.

#### Predictors of Policy Support to Reduce Flights

When regressing policy support to reduce air travel on the examined predictor variables, we found that efficacy beliefs that reduced aviation helps to mitigate climate change and intergenerational justice concerns were the strongest predictors, followed by perceived behavioral control to travel without flying, and a weaker pro-travel attitude. Low environmentally careless holiday attitude, subjective knowledge, age, and low education predicted policy support significantly but with weaker relationships. Global identity was not a significant predictor.

The positive relation between perceived efficacy of avoiding air travel as means to mitigate climate change and policy support resonates with similar findings that the perceived effectiveness of concrete policy measures predicts their support (Bergquist et al., 2022). Even though we asked about the effectiveness of the behavior change *per se* instead of policies encouraging this behavior change, it makes sense that perceiving the target behavior of policies to be an effective means to achieve a desired goal (in this case climate mitigation) facilitates policy support.

Previous research has further demonstrated that the perceived fairness of policies is a particularly strong correlate of policy acceptance (Bergquist et al., 2022). Fairness perception, in turn, might depend on justice concerns. This might be reflected in our result that concerns about justice, in this case intergenerational justice, were positively related to policy support in favor of reducing unfair climate impacts for future generations (even though we did not concretely ask about the fairness of policies from an intergenerational perspective).

Perceived behavioral control to travel without planes predicted policy support positively. It could be argued that people feel less personally affected by newly introduced restrictions of air travel if they believe to have sufficient alternatives and hence show higher support for these policies. This would support previous studies demonstrating a negative influence of perceived intrusiveness, namely personal costs, on support for policies (Huber et al., 2020). Unsurprisingly, people who agreed that everybody should be able to vacation wherever they want to in the world (pro-travel attitude) showed lower support for policies that aim at reducing air travel as this might restrict the possibility to reach certain remote destinations.

Despite a small positive bivariate correlation between global identity and policy support, this relation vanished when controlling for the other predictors in the regression model. This contrasts with the study by Loy et al. (2021). As argued for behavioral intentions, conflicting interests between international travel desires and environmental concerns could again play a role. Global identity became a positive predictor of policy support, when we removed intergenerational justice concerns from the model.

To summarize, perceived behavioral control to travel without flying and efficacy beliefs that reducing flights contributes to climate protection were the most relevant predictors of both, intentions and policy support to reduce flights. Moreover, a concern for intergenerational justice might motivate people to find alternatives for flying and to support aviation-related environmental policies.

#### Effect of Information on Intentions to Avoid Flights and Policy Support

Air travel is a domain highly intertwined with justice-related questions—from a global, intranational, and intergenerational point of view. Knowledge about environmental problems and related injustices (e.g., coming along with standards of living, specifically in the Global North) is considered a prerequisite for environmental action to be initiated (Frick et al., 2004; Jacobson et al., 2020). Thus, we tested whether providing additional information on environmental impacts and different inequalities linked to air travel influenced intentions to avoid flights and policy support to reduce air travel. We compared three types of information with a control group not receiving information: climate impact, global and intranational inequalities, and subsidies (implying intranational inequalities). We hypothesized that information on subsidies implying intranational inequalities might be particularly engaging for people with a strong national identity, while people with a strong global identity might be particularly receptive to information on not only intranational but also global inequalities.

None of the three information texts had an impact on intentions to avoid flights and policy support. In addition, we found neither an interaction effect between information on global inequality and global identity, nor between subsidies (implying intranational inequalities) and national identity. In sum, merely providing information on climate and justice-related aspects of air travel did not have an effect in our sample, neither on the individual behavioral dimension nor on policy support.

This finding resonates with psychological research that providing information tends to have only small effects if at all as long as it is not combined with, for example, action-enabling information (Frick et al., 2004). Moreover, the overall awareness of our sample might have already been quite high due to the public attention regarding problems of air travel raised by the Fridays for Future movement. Providing additional information might therefore not motivate engagement (anymore). Yet, it was surprising to us that especially global identity did not interact with the content of the textual information.

#### Use and Attractiveness of Different Long-Distance Transport Modes

According to our data, there seems to be a basic awareness and attention of the population to the connection between climate change and air travel (represented in subjective knowledge). Our data reveal that only a small part of the respondents fly on a regular basis. More than half of our respondents did not fly at all in the past year, at least within Europe. This finding is in line with other studies (Gössling and Humpe, 2020; Dütschke et al., 2022). Yet, travel time and price were the two criteria ranked as most important for choosing transport means among the majority of our respondents, whereas almost half ranked environmental friendliness last. Hence, the (infrastructural) availability and a subjectively positive evaluation of alternatives (e.g., in terms of travel time and price) could contribute to the promotion of sufficiency-oriented mobility. In terms of travel time, night trains might constitute a practical approach for traveling longer distances within Europe. This is supported by our finding that night trains were generally evaluated as an attractive form of travel but only some of the respondents had used them so far. A majority would like to see an increase in the number of night trains.

### Limitations and Future Research Directions

Several limitations of this research need to be discussed. First, most results are correlational and no conclusions on causality of the bivariate relationships between variables can be drawn. Future research should thus develop longitudinal and/or experimental designs to better establish the direction of possible effects.

Second, the operationalization of the assessed variables could be improved. Some scales were lacking internal consistency (e.g., pro-travel attitude). Furthermore, we used several one-item measures for reasons of practicality and questionnaire brevity. More extensive (pre-tested) scales would increase reliability and validity of the measurement. Concern for intergenerational justice was a central component of this study. As we did not find a tested scale, we developed items *ad hoc* based on theoretical considerations by Russell et al. (2003). Future work could take a step back and develop and validate a profound theoretically based scale. Still, internal consistency of our items was high after removing two items.

Third, there are several other factors that explain pro-environmental behavior (Bamberg and Möser, 2007; Klöckner, 2013). Specifically, social and personal norms might be relevant, even though, as described, research revealed mixed evidence for air-travel related behavior (Davison et al., 2014; Dütschke et al., 2022). Still, future studies could include them and help to disentangle inconsistencies.

Despite these limitations, our study also holds several strengths. First, while previous studies used rather selective samples (e.g., Loy et al., 2021), our results were generated with a heterogeneous sample mirroring diverse segments of the German population.

Second, air travel is a highly relevant individual behavior in terms of its climate impact which has received minor attention in environmental psychology research. Our study thus contributes to the little initial knowledge about people’s motivation to refrain from flying (Loy et al., 2021; Oswald and Ernst, 2021; Dütschke et al., 2022).

Third, concern for intergenerational justice has not received much attention in psychological research but seems especially relevant due to the long-term consequences of climate change for future generations. For example, research by Hauser et al. (2014) showed that people are willing to avoid overexploitation of resources for the benefit of future generations when resource use is democratically determined by vote. Syme et al. (2000) argued that perceiving intergenerational injustice impacts pro-environmental political compliance. Furthermore, Reese and Jacob (2015) found that valuing intergenerational justice of resource use was related to people’s intentions to act pro-environmentally. Our study adds the relevance of people’s concern for intergenerational justice for decision making in the context of air-traveling. Future research could examine how concerns for intergenerational justice emerge. For example, one could assume that having a child might induce parents to care more for environmental protection. Even though Milfont et al. (2020) found no relation between parenthood and environmental concern or changes in routines and standard of living, parents might constitute a suitable target group responsive for interventions that address the future state of the planet as basis for enabling well-being of their children. Future research could test whether experimentally evoked salience of intergenerational injustices of climate change leads to different outcomes for parents compared to childless adults.

Only a small share of the population takes planes on a regular basis and their environmental impact is outstandingly high. Future studies could thus focus specifically on frequent flyers, examining factors that explain the high frequency of taking planes. It would be particularly interesting to understand how injustices from which this group benefits are perceived by them. Moreover, other variables potentially explaining the intention to avoid flights might be further investigated. For example, the symbolic and social value of air travel could be examined as individual behavior is intertwined with and guided by social structures (O’Connor, 2015; Windsor, 2018; Nguyen-Van et al., 2021). Further research could also consider people in their political actions. Aviation-related public sphere behaviors like engaging in movements that promote alternative traveling styles could be analyzed. Public sphere behavior and niche movements can contribute to changing the mobility system and its regulative and economic frame (Geels, 2004).

Resonating with prior research on the relation between justice concerns and global identity (Reese et al., 2014), in our study, concern for intergenerational justice was correlated with global identity. This implies that people who identified themselves more with the world community also showed higher concerns for intergenerational justice than individuals with lower global identification. However, we only found weak correlations of global identity with intentions and policy support to reduce air travel. These relations turned insignificant when considering further predictors in the regression models. In case of behavioral intentions, global identity even became a negative predictor, even though the effect size was very small. Therefore, future research could dive deeper into examining potentially conflicting norms and values of individuals with high global identity in terms of flying.

People’s perceived behavioral control to be able to travel without planes and their efficacy beliefs to thereby contribute to climate protection were the strongest predictors of intentions and policy support to reduce air travel in our study. Therefore, future research should investigate ways to foster these control beliefs. In particular, we deem it interesting how structural changes (e.g., improvement of the train infrastructure, lower prices of sustainable mobility options) would impact people’s perception that giving up flights is possible.

### Practical Implications

In addition to a general reduction in (long-distance) travel, switching to (night) trains, especially for travel within Europe, constitutes a relevant way of reducing emissions. The existence of alternatives and the associated perception of control over (sustainable) travel options are central to promoting sustainable mobility. With this study, we demonstrated that the more people perceive to have enough alternatives for vacation without flying and that avoiding air travel helps mitigate climate change, the more they support aviation-regulating policy measures as well as individually intend to refrain from flying. This finding supports claims from environmental organizations and civil society (see e.g., Stay Grounded^1^) that expanding infrastructure for climate friendly alternatives, such as railway or cycling routes, and ceasing subsidies for carbon-intensive transport means can contribute to reducing air travel. A majority of our participants opted for an increase in night trains but there are other reasons besides availability that can be addressed to provide an attractive alternative travel option to air travel in the future (e.g., cost of tickets). In addition, opportunities could be given to encourage people to try out night trains and thereby gain experience (e.g., taster offers). In summary, (night) trains can make a decisive contribution to reducing emissions in transport, as both travel by car and by air can be substituted. By creating suitable framework conditions and infrastructure, this form of sufficiency-oriented mobility behavior can be promoted.

Traveling seems to have many advantages—among them possibilities for individual growth and personality development through international exchange (e.g., Phillips, 2019). However, there might be ways of becoming aware of other cultures and develop openness toward others without traveling by plane. As frequency and quality of contact with people during journeys might be more important than being frequently abroad *per se* (Loy et al., 2021), slow travel modes might be similarly or even better suitable. Showing opportunities for traveling on the ground (e.g., terran^2^) and setting an example for environmentally friendly travel behavior might establish new travel norms particularly for young people who have not yet become used to flying.

## Conclusion

Concerted, timely action is of uttermost importance for mitigating climate change. Air traffic already plays a strong role in driving climate change and is projected to grow—with only limited technical potential for decarbonizing this means of transport. Therefore, it is desirable to minimize the expansion of air traffic or even facilitate a reduction in affluent countries. Effective policies and behavioral change, especially among frequent flyers, can help to lower greenhouse gas emissions. For both, a positive evaluation and public support are indispensable.

We contribute to understanding people’s intentions and policy support to reduce air traveling and found that three aspects were most important in our sample: perceived behavioral control to travel without flying, beliefs in positive outcomes of flight avoidance, and intergenerational justice concerns. Therefore, we conclude that the mobility sector should facilitate attractive and accessible transport alternatives to planes. Moreover, the value of intergenerational justice needs to be conveyed in order to motivate people to reduce aviation as means to foster a livable future for new generations.

## Data Availability Statement

The datasets presented in this study can be found in online repositories. The names of the repository/repositories and accession number(s) can be found below: http://dx.doi.org/10.24406/fordatis/69.

## Author Contributions

JB conceptualized and designed the study, conducted the analysis, and interpreted the data. JB drafted the introduction, methods, results and the discussion section, and contributed to the theoretical background. AB contributed to the analysis and interpretation of the data. AB wrote parts of the theoretical background and discussion. LL contributed to the interpretation of the data and wrote parts of the introduction, theoretical background, results and discussion. All authors contributed to the manuscript revision, read, and approved the submitted version.

## Conflict of Interest

The authors declare that the research was conducted in the absence of any commercial or financial relationships that could be construed as a potential conflict of interest.

## Publisher’s Note

All claims expressed in this article are solely those of the authors and do not necessarily represent those of their affiliated organizations, or those of the publisher, the editors and the reviewers. Any product that may be evaluated in this article, or claim that may be made by its manufacturer, is not guaranteed or endorsed by the publisher.

## Footnotes

1
https://stay-grounded.org/

2
https://terran.eco/en/
