# Supplementary material for: Give Up Flights? Psychological Predictors of Intentions and Policy Support to Reduce Air Travel
Source: Front Psychol. 2022 Aug 4;13:926639. doi: 10.3389/fpsyg.2022.926639 (PMC9387255; doi:10.3389/fpsyg.2022.926639)
Supplement: Supplementary file 1 [file Table_1.docx]

Supplementary Material

Table S1. Variable descriptions.

| **Construct/ Variable** | **Item description** | **Descriptive** | |
| --- | --- | --- | --- |
|  |  | **Mean** | **Standard Deviation** |
| Intention to avoid flights | 1. I do not plan to fly within Germany in the next 12 months. 2. I plan to travel within Europe by means other than air in the next 12 months. 3. In the future, I will try to avoid flights in general.^1^ | 3.64 3.45 3.24 | 1.70 1.52 1.49 |
| Policy support | 1. Overall, I am in favor of policies being put in place to reduce air traffic as a whole. 2. Overall, I am in favor of political measures being taken to reduce domestic air traffic. 3. There should be a general ban on advertising air travel. 4. Domestic air travel should be made significantly more expensive. 5. Each person should receive a "CO_2_ flight budget", which can be spent per year and accumulated over the years (e. g. 2,000 km flight distance per year; if no flight for 5 years, 10,000 km are still available). 6. Flights should become more expensive the more a person flies. 7. Domestic flights should be banned. 8. A maximum number of domestic flights per year should be set for all airlines. | 3.40 3.58 2.56 3.39 2.91  3.01 2.62 3.15 3.30 | 1.36 1.33 1.40 1.40 1.44  1.48 1.35 1.43 1.00 |
| Pro-travel attitude | 1. Low-cost airlines have opened better opportunities for people to travel more regularly.^1^ 2. All people in Germany should have the opportunity to vacation wherever they want in the world. 3. It's important to me to take vacations and short breaks.^1^ | 3.41 3.73  3.82 | 1.21 1.18  1.06 |
| Holiday environ-mental attitude | I don't worry about the environment when I make decisions about my vacation travel. | 2.96 | 1.32 |
| Perceived behavioral control | I have enough options for a good vacation without having to fly. | 3.73 | 1.21 |
| Efficacy beliefs | Avoiding air travel helps fighting climate change. | 3.42 | 1.29 |
| Global identity | 1. Overall, being a citizen of the world is an important part of how I see myself. 2. I feel strongly connected to other people in this world. | 3.39 3.10 3.25 | 1.14 1.11 0.98 |
| Intergenerational justice concerns | 1. We should take up all efforts to secure the livelihood of future generations. | 4.03 | 0.96 |
|  | 1. I am very concerned that future generations will feel the climate and environmental problems caused by previous generations | 3.58 | 1.16 |
|  | 1. Each generation must find its own way to deal with the livelihoods it finds. *(inverse)* | 3.29 | 1.22 |
|  | 1. I will not reduce my standard of living to reduce the climate and environmental impact for future generations^1^ *(inverse)* | 2.55 | 1.12 |
|  | 1. I feel it is unfair for future generations to have to deal with the climate and environmental consequences of our lifestyle^1^ | 3.72  2.83 | 1.13  0.69 |
| Subjective knowledge | I could spontaneously explain to a friend what air travel has to do with climate change. | 3.20 | 1.25 |
| National Identity | 1. The nation I belong to is an important reflection of who I am. 2. Overall, my nation has very little to do with how I feel*. (inverse)*^2^ 3. Overall, belonging to this nation is an important part of how I see myself. | 3.07 2.76  3.36 3.22 | 1.17 1.13 1.13 1.01 |

*Note*. All items were assessed on a five-point Likert scale ranging from 1 (*do not agree at all*) to 5 (*agree very much*). ^1^For content reasons, only this item was used for further statistical analysis, as it best reflects the general willingness to avoid flights. ^2^This item was left out from further analysis due to lowering internal consistency (Cronbach’s *α* < 0.5).

Table S2. Results of linear multiple regression of intentions to avoid flights.

| Predictor | *B* | *SE* | β | *t* |
| --- | --- | --- | --- | --- |
| Age | .01 | .00 | .11 | 6.86*** |
| Gender (dummy) | .07 | .05 | .02 | 1.35 |
| Income | -.00 | .00 | -.01 | -.77 |
| High education (dummy) | -.34 | .05 | -.11 | -6.61*** |
| Pro-travel attitude | -.21 | .02 | -.17 | -9.96*** |
| Environmentally careless holiday attitude | -.08 | .02 | -.07 | -4.31*** |
| Perceived behavioral control | .43 | .02 | .35 | 20.31*** |
| Efficacy beliefs | .29 | .02 | .25 | 13.44*** |
| Global identity | -.08 | .03 | -.05 | -2.72** |
| Intergenerational justice | .29 | .04 | .14 | 6.71*** |
| Subjective knowledge | -.01 | .02 | -.01 | -.29 |
| Information climate (dummy) | -.13 | .07 | -.04 | -2.01* |
| Information inequality (dummy) | -.13 | .19 | -.04 | -.67 |
| Information subsidies (dummy) | -.06 | .19 | -.02 | -.33 |
| National identity | .02 | .03 | .01 | .60 |
| Information inequality * global identity | .01 | .06 | .01 | .23 |
| Information subsidies * national identity | -.02 | .05 | -.02 | -.35 |

*Note*. *R²_corr_* = .43 (*p* < .01). * *p* < .05, ***p* < .01, ****p* < .001.

Table S3. Results of linear multiple regression of policy support to reduce flights.

| Predictor | *B* | *SE* | β | *t* |
| --- | --- | --- | --- | --- |
| Age | .00 | .00 | .06 | 3.66*** |
| Gender | .01 | .03 | .01 | .34 |
| Income | -.00 | .00 | -.01 | -.36 |
| High education (dummy) | -.08 | .03 | -.04 | -2.41* |
| Pro-travel attitude | -.15 | .01 | -.17 | -10.13*** |
| Environmentally careless holiday attitude | -.07 | .01 | -.09 | -5.32*** |
| Perceived behavioral control | .14 | .01 | .17 | 9.73*** |
| Efficacy beliefs | .22 | .02 | .29 | 15.24*** |
| Global identity | .00 | .02 | .00 | .11 |
| Intergenerational justice | .38 | .03 | .26 | 12.88*** |
| Subjective knowledge | .05 | .01 | .07 | 3.73*** |
| Information climate (dummy) | .05 | .04 | .02 | 1.09 |
| Information inequality (dummy) | -.04 | .13 | -.02 | -.34 |
| Information subsidies (dummy) | -.04 | .13 | -.02 | -.33 |
| National identity | -.01 | .02 | -.01 | -.39 |
| Information inequality * global identity | .00 | .04 | .01 | .12 |
| Information subsidies * national identity | .03 | .04 | .04 | .83 |

*Note*. *R²_corr_* = .45 (*p* < .001). * *p* < .05, ***p* < .01, ****p* < .001.
